# Dietary Modulation of the Immune Function: Direct and Microbiota-Dependent Effect

**DOI:** 10.3390/nu14091957

**Published:** 2022-05-07

**Authors:** Francisco J. Pérez-Cano

**Affiliations:** 1 Department of Biochemistry and Physiology, Faculty of Pharmacy and Food Science, University of Barcelona, 08028 Barcelona, Spain; franciscoperez@ub.edu; Tel.: +34-9-340-24505; 2 Nutrition and Food Safety Research Institute (INSA-UB), 08921 Santa Coloma de Gramenet, Spain

Diet is critical in maintaining optimal immune function. Extensive research has demonstrated the immunomodulatory properties of particular nutrients. In this regard, some dietary components are able to modulate the immune response by interacting directly with the mucosal and systemic immune cells, activating receptors (e.g., vitamins) or changing membrane properties or modulating gene expression (e.g., fatty acids). However, some nutrients may influence immune function indirectly, after being metabolized by the microbiota, either by generating new active components (e.g., polyphenol metabolites, short-chain fatty acids derived from fiber) or just by shaping microbial composition and functionality (e.g., probiotics and prebiotics), which in turn, will affect the immune response. The impact of these components is not only located at the intestine, where the first contact nutrients-host takes place, but can be extended to the systemic compartment and linked to other organic systems.

This Special Issue of Nutrients “Dietary Modulation of the Immune Function: Direct and Microbiota Dependent Effect” includes seven publications, one of them as an original publication [1] but also six reviews highlighting the importance of key dietary components on the immune system and its relation to the microbiota. These latter publications are focused on specific situations of the diet–body interaction in the human life: early life and immunodevelopment (breast milk) [2], in the context of health in different animal species (fructans) [3] or connected to other parts of the body such as the gut–brain axis or the gut–breast axis [4,5]. In addition, the role of polyphenols with microbiota and immunity in situations with altered immune response such as exercise or inflammatory and metabolic diseases [6,7] has also been reviewed in this topic.

Starting in early life, it is well known that breast milk is the first food that infants receive, and that is produced by the mother’s breasts in order to both nourish and help them with protection from disease. The review by Rio-Aige et al. [4] remarks that the concentrations and proportions of some immune components, such as the immunoglobulins (defined as breast milk immunoglobulinome), are influenced by intrinsic factors—such as the sampling time and quantification technique—but also by extrinsic factors—such as lifestyle and environment—which in turn have an influence on the intestinal microbiota composition. This fact also links to the revision of Rodríguez et al. [5], which focused specifically on the axis linking the maternal gut (including microbiota), the breast and the infant gut. It describes how this interaction is critical for correct infant growth and development.

The first prebiotic and probiotic compounds that an individual receives are also provided by breast milk and are involved in this early process of immune maturation and in driving the postnatal infant’s microbiota. Later in life, both types of microbial modulators are still key elements in the dietary modulation of immunity. For instance, in this Special Issue, a review exploring the role of fructans in immunity has been included [3]. This article provides an overview of the relevant in vitro and in vivo studies of both the β-2,6 fructans (from plant origin) and levan (from microbial origin) on immunity showing many effects but requiring additional studies.

Besides the promising effects of the above prebiotics, the intervention with probiotics seems to be more effective in several diseases. In this line, the anti-inflammatory mechanisms of three probiotic strains, *Bifidobacterium breve* CNCM I-4035, *Lactobacillus paracasei* CNCM I-4034 and *Lactobacillus rhamnosus* CNCM I-4036, are aligned to their ability to modulate macrophage polarization and to decrease their pro-inflammatory gene expression and leukocyte infiltration in the liver in a model of non-alcoholic fatty liver disease (NAFLD) [1].

Besides these classical microbial modulators, the diet provides polyphenols, which are regularly ingested in small quantities and include, among others, flavonoids and a particular type of them, the flavanols. The systematic review by Ruiz-Iglesias et al. [6] explored the role of these components in exercise performance in association with immune function. This research found that less than 40% of studies evaluating exercise performance following flavonoid supplementation reported improvement and that only a minority of them evaluated their potential in the affectation of the immune response associated with exercise. In addition, Martín and Ramos [7] reviewed the complex interaction of flavanols with gut microbiota, immunity and inflammation in the context of diabetes, obesity and metabolic syndrome. They displayed many mechanisms involved observed by in vitro and in vivo approaches, including those dependent of microbiota metabolism, but finally concluded that many pathways and connections need to be further explored.

Finally, this Special Issue ends with a review by Karakan et al. [7], which focused on the gut–brain axis and the role of microbiota in this complex interaction of systems. It showed that both human and animal studies provide evidence for a causal relationship between gut microbiota and brain functions and the role of antibiotics affecting it.

Overall, the present Special Issue provides a summary of the progress on the diet–microbiota–immunity interaction, going in depth into the participating mechanisms. Further research is encouraged to keep exploring the effects and mechanisms of the above foods (breast milk) and bioactive compounds (pre- and probiotics, polyphenols) and many others not included here (e.g., vitamins, PUFA, etc.) with the aim of establishing their high importance in health and disease.
